# A Simple Evaluation of Soil Quality of Waterlogged Purple Paddy Soils with Different Productivities

**DOI:** 10.1371/journal.pone.0127690

**Published:** 2015-05-21

**Authors:** Zhanjun Liu, Wei Zhou, Jialong Lv, Ping He, Guoqing Liang, Hui Jin

**Affiliations:** 1 Ministry of Agriculture Key Laboratory of Crop Nutrition and Fertilization, Institute of Agricultural Resources and Regional Planning, Chinese Academy of Agricultural Sciences, Beijing 100081, China; 2 College of Resource and Environmental Science, Northwestern University of A & F, Yangling 712100, China; 3 Institute of Agricultural Resources and Economy, Shanxi Academy of Agricultural Sciences, Taiyuan, 030006, China; Old Dominion Univ., UNITED STATES

## Abstract

Evaluation of soil quality can be crucial for designing efficient farming systems and ensuring sustainable agriculture. The present study aimed at evaluating the quality of waterlogged purple paddy soils with different productivities in Sichuan Basin. The approach involved comprehensive analyses of soil physical and chemical properties, as well as enzyme activities and microbial community structure measured by phospholipid fatty acid analysis (PLFA). A total of 36 soil samples were collected from four typical locations, with 12 samples representing high productivity purple paddy soil (HPPS), medium productivity purple paddy soil (MPPS) and low productivity purple paddy soil (LPPS), respectively. Most measured soil properties showed significant differences (P ≤ 0.05) among HPPS, MPPS and LPPS. Pearson correlation analysis and principal component analysis were used to identify appropriate soil quality indicators. A minimum data set (MDS) including total nitrogen (TN), available phosphorus (AP), acid phosphatase (ACP), total bacteria (TB) and arbuscular mycorrhizal fungi was established and accounted for 82.1% of the quality variation among soils. A soil quality index (SQI) was developed based on the MDS method, whilst HPPS, MPPS and LPPS received mean SQI scores of 0.725, 0.536 and 0.425, respectively, with a ranking of HPPS > MPPS > LPPS. HPPS showed relatively good soil quality characterized by optimal nutrient availability, enzymatic and microbial activities, but the opposite was true of LPPS. Low levels of TN, AP and soil microbial activities were considered to be the major constraints limiting the productivity in LPPS. All soil samples collected were rich in available N, K, Si and Zn, but deficient in available P, which may be the major constraint for the studied regions. Managers in our study area should employ more appropriate management in the LPPS to improve its rice productivity, and particularly to any potential limiting factor.

## Introduction

Waterlogged paddy soil is a common soil type associated with low productivity, and occupies a large area putting constraints on crop production in southwest China. Purple soil (FAO, Regosol) is widely distributed in this area, where it is susceptible to soil erosion and nutrient loss due to terrain disturbance by humans [1]. Therefore, numerous previous studies mainly focused on nutrient loss and its associated factors [2, 3]. An improved knowledge of soil quality assessment is important for developing appropriate soil anti-degradation measures and design management plans [4]. This is particularly important as little is known about soil quality status of waterlogged paddy soils, particularly in the purple soil region.

Soil quality has received more attention recently in response to an increasing public interest in sustainability and soil resources [5]. However, there is no established methodology so far to characterize soil quality based on a universal set of indicators [6]. Selecting representative variables is critical for soil quality evaluation [7], which should combine soil physical, chemical and biological properties [8]. At present, a universal set of the main indicators to evaluate soil quality is still the subject of debate [9]. While numerous studies on soil quality evaluation have concentrated on soil physical and chemical properties [10,11], biological variables can also be important and have been increasingly used as indicators of soil quality owing to their rapid response and high sensitivity to changes in soil management [12,13]. These studies illustrate the effects of specific agricultural management practices on soil quality. However, little information is available in the literature that evaluates soil quality aiming at any special soil type in terms of productivity on a regional scale [4,14,15], particularly to the purple paddy soil. In this way, a comprehensive assessment of soil quality integrating soil physical, chemical and biological properties would be greatly desired.

An accurate evaluation of soil quality requires analyzing a large number of soil parameters [16]. Usually, a minimum data set (MDS) was established based on a careful selection [12,17], and then a soil quality index (SQI) was also increasingly developed to quantify and compare the quality status of soils related to different management practices [11,15]. However, many previous studies typically did not provide crop-yield data [2,18,19] or looked further into the relationship between SQI and yield [10,17], thus making their results likely have little biological significance.

In our study, we examined soil physical, chemical and biological properties with a goal to provide a comprehensive assessment of soil quality for different productive waterlogged paddy soils in the acidic purple soil region. Our objectives were to: (*i*) establish an MDS for soil quality evaluation; (*ii*) develop a SQI to quantify soil quality status; and (*iii*) identify the limiting factors associated with the crop productivity of waterlogged acidic purple paddy soils.

## Materials and Methods

### Study area

The measurements were conducted only on the most representative areas of waterlogged purple paddy soils. Four typical locations, Tongliang (106°04' E, 29°46' N), Beibei (106°23' E, 29°54' N), Luxian (105°23' E, 29°11' N) and Tianxing (105°19' E, 29°06' N) were selected accounting for the optimal analytical costs. These four areas are situated in Sichuan Basin, southwest China. The selected paddy fields are private lands, and the study was carried out following the permission of each land ower. A subtropical monsoon climate prevails in those regions, with a mean annual temperature and mean annual precipitation of 17.0°C and 1000 mm, respectively. Precipitation varies in each month, and 70% occurs from May to September. The soil type of the study area is the acidic purple paddy soil, and land use is dominantly arable with a single-harvest rice per year. The parent material is purple sandstone, and the mineral compositions include vast hydrous mica and slight vermiculite.

According to the mean annual rice yield over the past five years, the selected paddy fields were divided into three classes with high (> 7500 kg ha^–1^, HPPS), medium (6000–7500 kg ha^–1^, MPPS) and low (<6000 kg ha^–1^, LPPS) productivity. Based on farmers' surveys, conventional fertilization focused on mineral fertilizers, and fertilizer types were CO(NH_2_)_2_, Ca(H_2_PO_4_)_2_ and KCl for N, P, and K, respectively. Similar fertilization was employed in HPPS and MPPS, with the mean rates of 155 kg N ha^-1^ (2:1 basal: topdressing), 60 kg P_2_O_5_ ha^-1^ (100% basal) and 80 kg K_2_O ha^-1^ (1:1 basal: opdressing), respectively, and mean rates of 125 kg N ha^-1^ (2:1 basal: topdressing), 60 kg P_2_O_5_ ha^-1^ (100% basal) and 80 kg K_2_O ha^-1^ (1:1 basal: topdressing) were applied to the LPPS. One tillage operation was conducted before the cropping season, and the sampling areas were considered typical waterlogged purple paddy soil regions because of their concentrated distributions, similar weather conditions, cropping systems, agricultural management (i.e. fertilization, tillage regime) and productivity levels.

### Soil sampling

**Fig 1:**
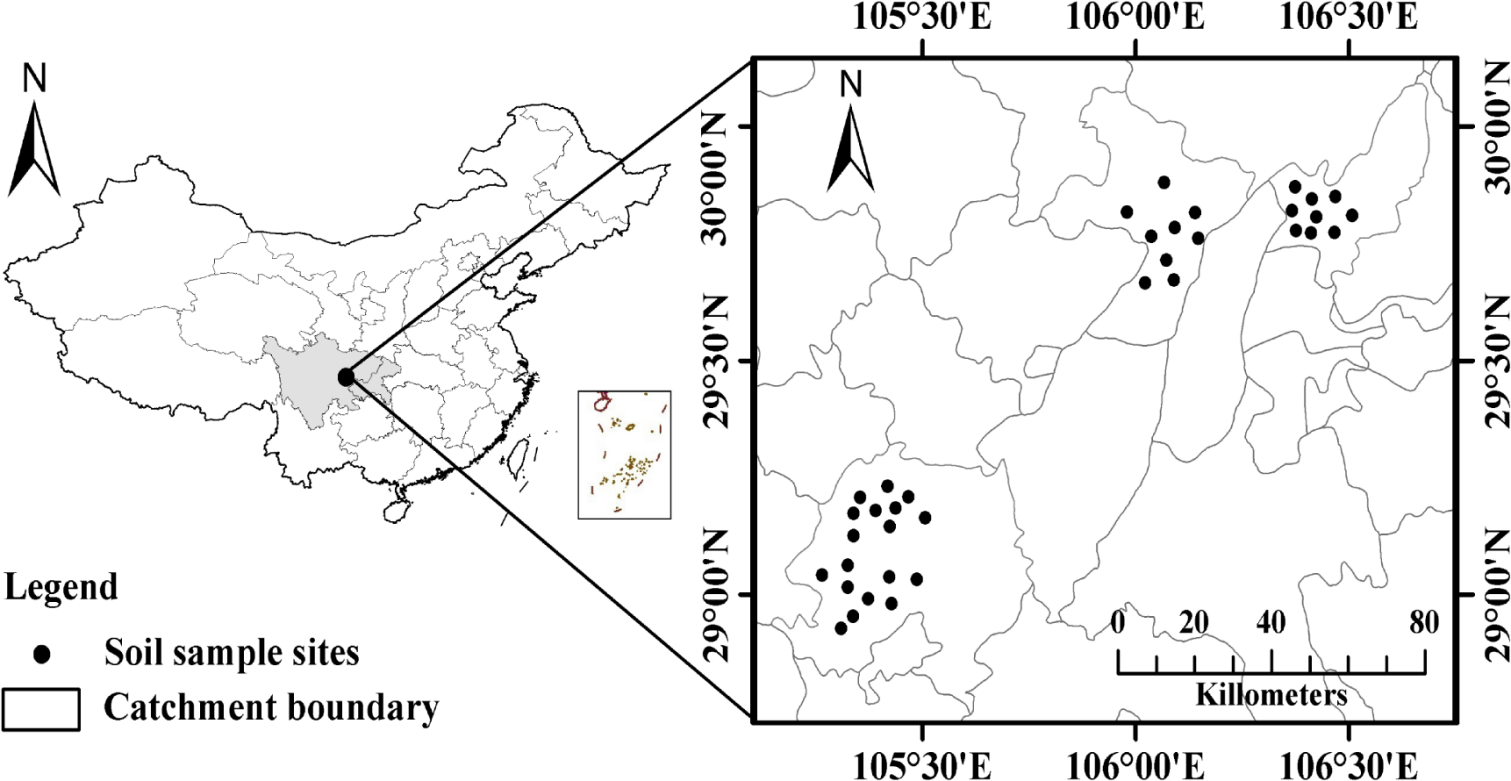
Geographic locations of soil sampling sites.

Based on the distribution of waterlogged purple paddy soils and their productivities, 36 waterlogged paddy fields were selected as sampling sites, and their geographical locations are shown in Fig 1. In each selected waterlogged paddy field, ten cores (5.0 cm diameter) were collected randomly from a soil depth of 0–15 cm (plow layer) and well-mixed to form a composite sample during jointing stage in May 2012. In each typical location, three composite soil samples were collected to represent each productivity class of waterlogged paddy soil. Those samples were immediately transported to the laboratory, and one sub-sample was air-dried at room temperature for physical and chemical analysis, one sub-sample was stored at 4°C for biochemical analysis and the last sub-sample was freeze-dried prior to being stored at −18°C for phospholipid fatty acids (PLFA) analysis.

### Soil analysis

#### Physico-chemical properties

Soil aggregate stability (SAS) was measured using the wet sieving method [20]. Soil texture, bulk density (BD), total nitrogen (TN), available nitrogen (AN), available phosphorus (AP), soil organic matter (SOM), pH (soil/water 1:2.5), available potassium (AK), available zinc (AZn, 0.01 M hydrochloric acid extraction), available silicon (ASi, 0.025 M citric acid extraction) and cation exchange capacity (CEC) were determined using the methods described by Page et al. (1982) [21].

#### Enzyme activity

The activities of β-glucosidase, acid phosphatase and peroxidase were measured according to DeForest (2009) [22], and arylsulfatase activity was essayed as described by Giacometti et al. (2014) [23], whilst urease and dehydrogenase activities were determined based on Alef and Nannipieri (1995) [24].

#### Microbial community structure

Microbial community structure was determined using PLFA analysis as described by Wu et al. (2009) [25]. Concentrations of PLFAs were calculated as nmol g^−1^ and mol%. PLFA bio-indicators were selected according to the procedure of Moeskops et al. (2010) [26], using only the PLFAs clearly identified by GC-FID: fatty acids iC15:0, aC15:0, iC16:0, iC17:0 and aC17:0 were used as biomarkers for Gram-positive bacteria (G+) and C16:1ω7c, C18:1ω7c and cyC17:0 for Gram-negative bacteria (G−). The sum of G+, G−, C:15, C17:0 and cyC19:0ω11,12c was assumed to represent the total bacterial community, and the sum of 10MeC16:0 and 10MeC18:0 was regarded as an indicator of actinomycetes. The fatty acids C18:2ω6,9c and C16:1ω5c were used as biomarkers for fungi and arbuscular mycorrhizal fungi (AMF), respectively.

### Soil quality evaluation method

#### Optimizing selection of a minimum data set (MDS)

According to the indicator selection method described by Li et al. (2013) [17], those soil properties that did not show a significant Pearson correlation (P < 0.05) with rice yields were eliminated from the data list. A principal component analysis (PCA) was conducted with the remaining soil variables to select the most appropriate indicators. Only principal components (PCs) with eigenvalues of ≥1 and which explained at least 5% of the variation in the data were considered [11,27]. Within each principal component (PC) only highly weighted factors (i.e. with absolute values within 10% of the largest factor loading) were retained for the MDS [28]. When more than one variable was retained in a PC, each was considered important and was considered in the MDS if the two considered values were not correlated (r < 0.60) [18]. Among well-correlated variables within a PC, the variable with the highest correlation sum was selected for the MDS [28].

#### Weight assignment and indicator scoring

MDS variables were included to another PCA, and the weight for each indicator was calculated by its communality and was equal to the ratio of its communality with the sum of communalities of all MDS indicators [29].

Because of different indicator units, a standard scoring function (SSF) was used to score all MDS variables using each indicator method, and the detailed scoring functions were the same as those used by Qi et al. (2009) [14]. For the MDS variables without a certain threshold value (the optimum), every observation was normalized using a linear scoring function [30]. For “higher is better” indicators, each observation was divided by the highest observed value and thus the highest observed value received a score of 1. For “lower is better” indicators, the lowest observed value (in the numerator) was divided by each value (denominator) and thus the lowest observed value received a score of 1. For those indicators which were neither higher is better nor lower is better, the values were scored as “higher is better” up to a threshold value and then scored as “lower is better” above the threshold value [10,30].

#### Developing an SQI

After the MDS indicators were weighted and scored, the SQI was calculated using the following equation described by Doran and Parkin [31]:

$$SQI=\sum_{i=1}^{n}Wi\times Si$$ (1)

where Wi is the assigned weight of each indicator, Si is the indicator score, and n is the number of variables in the final MDS.

### Data analysis

Data were subjected to statistical analysis using SPSS 18.0 (IBM Corporation). One-way analysis of variance (ANOVA) was used to test all the parameters, and the least significant difference (LSD) method at the probability level of 0.05 was used to separate mean difference of the soil attributes.

## Results and Discussion

### Soil physico-chemical attributes

All measured soil physical and chemical properties significantly differed between HPPS, MPPS and LPPS with an exception of available N (Table 1). Relatively lower bulk density (BD) was observed in HPPS, while LPPS had significantly higher BD that may adversely affect soil nutrient circulation and rice root growth [4]. Rice husk biochar may be particularly necessary in LPPS because of its beneficial amendment for improving soil poor physical characteristics such as lowering soil bulk density [11,32].

**Table 1:** Summary statistics of measured soil physical and chemical properties of high- (HPPS), medium- (MPPS) and low- (LPPS) productivity purple paddy soils (mean ± standard deviation and range of variation).

Soil parameters	HPPS (n = 12)		MPPS (n = 12)		LPPS (n = 12)	
	Mean	Range	Mean	Range	Mean	Range
Bulk density (g cm^-3^)	1.15±0.06 c	1.05–1.25	1.23±0.05 b	1.15–1.30	1.30±0.03 a	1.27–1.37
pH	4.73±0.41 b	4.28–5.50	4.94±0.25 b	4.59–5.44	5.46±0.48 a	4.64–5.95
Soil organic C (g kg^-1^)	27.3±3.06 a	23.4–33.3	20.7±4.52 b	16.6–29.3	20.6±3.09 b	16.9–26.2
Total N (g kg^-1^)	1.03±0.09 a	0.90–1.18	0.90±0.09 b	0.74–1.09	0.84±0.07 b	0.75–0.98
Available N (mg kg^-1^)	218±33.3 a	159–256	227±32.8 a	172–265	229±34.9 a	160–275
Available P (mg kg^-1^)	8.10±2.02 a	5.13–11.4	7.10±1.32 a	5.34–8.99	4.70±1.70 b	1.67–6.71
Available K (mg kg^-1^)	157±34.5 a	114–217	107±56.8 b	76.8–201	95.4±31.9 b	67.8–152
CEC (cmol kg^-1^)	12.9±3.39 c	8.28–18.8	16.0±3.72 b	11.3–21.7	20.4±1.30 a	18.0–21.9
Available Si (mg kg^-1^)	144±38.3 a	101–187	116±14.4 b	94.7–138	107±6.26 b	95.6–116
Available Zn (mg kg^-1^)	5.84±0.46 a	5.02–6.46	4.65±1.25 b	3.04–6.48	3.98±1.01 b	3.00–5.58

Means for the same property with different letters indicate significant differences at p ≤ 0.05.

Soil pH values varied from 4.40 to 5.87, as typical for acidic purple paddy soils. Soil pH showed lower values in HPPS and MPPS compared to LPPS, which were lower by 0.7 and 0.5, respectively. This is probably due to the relatively greater application of inorganic N fertilizer in HPPS and MPPS. This is consistent with the conclusion that recent soil acidification in China has resulted mainly from high N fertilizer inputs [33]. Soil acidification can result in nutrient deficiency such as K, Na, Ca and Mg and thus decrease crop production [34]. Rice straw addition beneficial for pH improvement should be applied to mitigate soil acidification [35], and the widely used N fertilizer type of urea could be replaced by (NH_4_)_2_SO_4_ because of its capacity of inhibiting the increase of ammonia-oxidizing bacteria and thus decreasing nitrification and soil acidification [36]. Soil organic carbon (SOC) plays a pervasive role in ensuring soil fertility and promoting soil ecological functions [37]. MPPS and LPPS had SOC contents without any significant differences, in contrast to HPPS characterized by larger SOC. The mean SOC showed a remarkable increase compared to the value of 12 g kg^-1^ obtained by SSSC (1996) [38]. In the last twenty years, an increasing number of farmers have restored rice straw to paddy fields, thereby contributing to the rapid increase of SOC [39].

Purple paddy soil is characterized by low TN content, and only 0.75 g kg^-1^ was observed in SSSC (1996) [38]. Table 1 points to significant differences among HPPS, MPPS and LPPS, with their mean contents of TN typically higher than 0.75 g kg^-1^, and which increased by 37.3%, 20.0% and 12.0%, respectively. This is inconsistent with the findings of Wang et al. (2009) [2], who concluded that the remarkable increase in TN may be attributable to the continued application of N fertilizer. In addition, soil available N (AN) varied from 159–275 mg kg^-1^, which is substantially higher than the threshold value of 100 mg kg^-1^ [40], while no statistically significant differences were observed among HPPS, MPPS and LPPS. Our results indicate that the rate of basic N fertilizer may be overused and, therefore, the basal: topdressing ratio (2:1) needs adjustment to make N fertilizer application more timed to coincide with the period of rapid N uptake by plants.

No significant differences were observed for available P between HPPS and MPPS, but their mean contents were significantly higher than LPPS (i.e. by 72.3% and 51.1%, respectively). Although the total input of P fertilizer was applied as basal, mean contents of AP were all lower than the critical value of 10 mg P kg^-1^ [41], indicating a serious deficiency of AP. This may be related to the low soil temperature during the seedling stage that adversely affects P availability [42]. Therefore it seems that the deficiency in soil P may be one of the most important constraints limiting rice growth. It is recommended that straw mulch is carried out during winter fallow season to improve soil optimum temperature and thus increase P bioavailability [43]. He (2003) reported that the effect of K application on increasing the crop productivity in purple soil was not significant when soil AK was over 80 mg kg^-1^ [40]. Our whole study area was sufficient in AK because their mean contents were all higher than the critical threshold value. As suggested by Li (1991) the sufficient AK may be attributable to inheriting high K from the parent materials of purple soils [44].

CEC can be a vital predictor of soil quality [45] and has been assessed as an indicator of soil erosion and degradation [46]. Significant differences were found among the different productivity classes of waterlogged purple paddy soils, giving ranking of HPPS < MPPS < LPPS (Table 1). The result is inconsistent with the observation of Tesfahunegn et al. (2011) [4], who concluded that soils with high productivity are usually characterized by high CEC. Soil CEC is related to its parent material [46], which is why serious soil erosion has occurred in HPPS and MPPS probably due to their significant reduction compared to LPPS. On the other hand, our studied paddy fields are located in hillslopes, and the concentrated precipitation (from May to October) could lead to more severe erosion and nutrient loss for HPPS and MPPS beacuse of their relative high-elevations [2]. This supports the previous conclusion and accounts for the notable low levels of CEC in HPPS. Black carbon, which has been demonstrated to be beneficial in increasing CEC [47], seemed particularly necessary in HPPS.

As shown in Table 1, the studied paddy fields were sufficient in soil available silicon (ASi), because their concentrations were all higher than the critical value of 60 mg Si kg^-1^ [48]. Similarly, the whole study area presented abundance of available zinc (AZn) whose concentrations were always higher than the critical value of 1.0 mg Zn kg^-1^ [49], although the contents of AZn in MPPS and LPPS were typically lower than those in HPPS. This is consistent with the finding of Liu (1994) [50], who reported that AZn is not a limited nutrient in acidic purple soils. And soil AZn abundance might be attributable to their parent materials and frequent inputs coupled to the application of mineral fertilizers [51].

### Enzyme activities

Enzyme activities play an essential role in nutrient cycling, changes in soil quality and the degree of soil degradation due to their sensitivities to small changes in soils [52]. In our study, six enzymes were measured to estimate the microbial activity (dehydrogenase, peroxidase), C cycle (β-Glucosidase), N cycle (urease), P cycle (acid phosphatase) and S cycle (arylsulphatase). In Table 2 summarized are the statistics of soil enzymatic activities used as soil quality indicators in all of the waterlogged purple paddy fields. Significant differences were observed among HPPS, MPPS and LPPS for all measured soil enzymes. Urease, dehydrogenase, β-glucosidase, arylsulphatase, acid phosphatase and peroxidase were significantly lower in LPPS than in HPPS (i.e. by 42.0%, 25.9%, 42.6%, 49.1%, 26.6% and 30.3%, respectively). Urease and β-glucosidase were lower by 27.1% and 28.8% in LPPS compared to the MPPS, respectively; but no significant difference was found between LPPS and MPPS with respect to dehydrogenase, acid phosphatase, arylsulphatase and peroxidase. The amount of dehydrogenase and acid phosphatase in MPPS was not significantly different than in HPPS, while the activities of urease, β-glucosidase, arylsulphatase and peroxidase were typically lower in MPPS than HPPS (i.e. by 20.2%, 19.5%, 28.1% and 27.1%, respectively). Overall, HPPS presented high enzymatic activities, indicating a substantial capacity of nutrient supply and a better soil quality. Opposite was true for LPPS where application of fly ash [53] or organic amendment [54] could be beneficial to maintaining nutrient balance and increasing soil enzyme activities.

**Table 2:** Summary statistics for measured enzymes of high- (HPPS), medium- (MPPS) and low- (LPPS) productivity purple paddy soils (mean ± standard deviation and range of variation).

Soil parameters	HPPS (n = 12)		MPPS (n = 12)		LPPS (n = 12)	
	Mean	Range	Mean	Range	Mean	Range
Urease (mg NH_4_^+^-N kg^-1^ h^-1^)	20.8±2.95 a	16.8–27.2	16.6±4.49 b	11.7–27.1	12.1±2.41 c	7.49–15.5
Dehydrogenase (μg TPF g^-1^)	148±52.6 a	73.9–241	126±38.3 ab	75.2–180	110±37.6 b	45.4–159
β-glucosidase (nmol g^-1^ h^-1^)	128±15.3 a	102–149	103±32.7 b	51.4–148	73.3±24.8 c	32.2–106
Arylsulfatase (nmol g^-1^ h^-1^)	7.48±2.88 a	4.02–11.2	5.38±2.24 b	2.25–9.08	3.81±1.01 b	2.58–5.92
Acid phosphatase (nmol g^-1^ h^-1^)	521±103 a	385–672	433±156 ab	172–585	382±136 b	207–534
Peroxidase (nmol g^-1^ h^-1^)	8.82±1.28 a	6.34–10.6	6.43±2.18 b	2.67–10.0	6.15±2.43 b	2.80–12.1

Means for the same property with different letters indicate significant differences at p ≤ 0.05.

### Microbial community structure

PLFA analysis showed its total concentrations ranging from 19.7 to 52.6 nmol g^-1^, and the selected microbial groups all presented statistically significant differences among HPPS, MPPS, and LPPS (Table 3). Compared to the HPPS which was chosen as a benchmark for soil quality, the concentrations of total PLFAs, Gram-positive bacteria, Gram-negative bacteria, total bacteria, actinomycetes, fungi and AMF were significantly lower in LPPS (i.e. by 36.0%, 30.3%, 45.2%, 37.1%, 32.9%, 37.8% and 46.6%, respectively); and were also significantly lower in MPPS (i.e. by 35.6%, 28.1%, 43.6%, 34.7%, 38.3%, 21.9% and 32.9%, respectively). With the exception of fungi, MPPS contained levels of total PLFAs and the selected marker PLFAs similar to those of LPPS.

**Table 3:** Concentrations of total and marker PLFAs (mean±standard deviation and range of variation, nmol g^–1^ dry soil).

Soil parameters	HPPS (n = 12)		MPPS (n = 12)		LPPS (n = 12)	
	Mean	Range	Mean	Range	Mean	Range
Total PLFAs	38.1±8.13 a	26.7–52.6	24.6±3.96 b	19.7–31.9	24.4±3.23 b	20.9–31.3
Gram-positive	7.52±1.63 a	4.81–9.92	5.41±0.82 b	4.00–6.75	5.25±0.71 b	4.24–6.59
Gram-negative	7.66±1.61 a	5.87–10.8	4.32±0.68 b	3.15–5.30	4.20±0.57 b	3.24–5.09
Total bacteria	16.2±1.41 a	14.3–18.3	10.5±1.69 b	7.60–12.7	10.2±1.13 b	8.41–12.4
Actinomycetes	3.79±1.40 a	1.86–5.93	2.34±0.31 b	1.92–2.82	2.54±0.72 b	1.57–3.70
Fungi	1.12±0.10 a	0.94–1.29	0.87±0.22 b	0.52–1.21	0.70±0.15 c	0.48–0.89
AMF	0.96±0.30 a	0.73–1.54	0.64±0.14 b	0.47–0.90	0.51±0.11 b	0.37–0.72

Means for the same property with different letters indicate significant differences at p ≤ 0.05.

**Fig 2:**
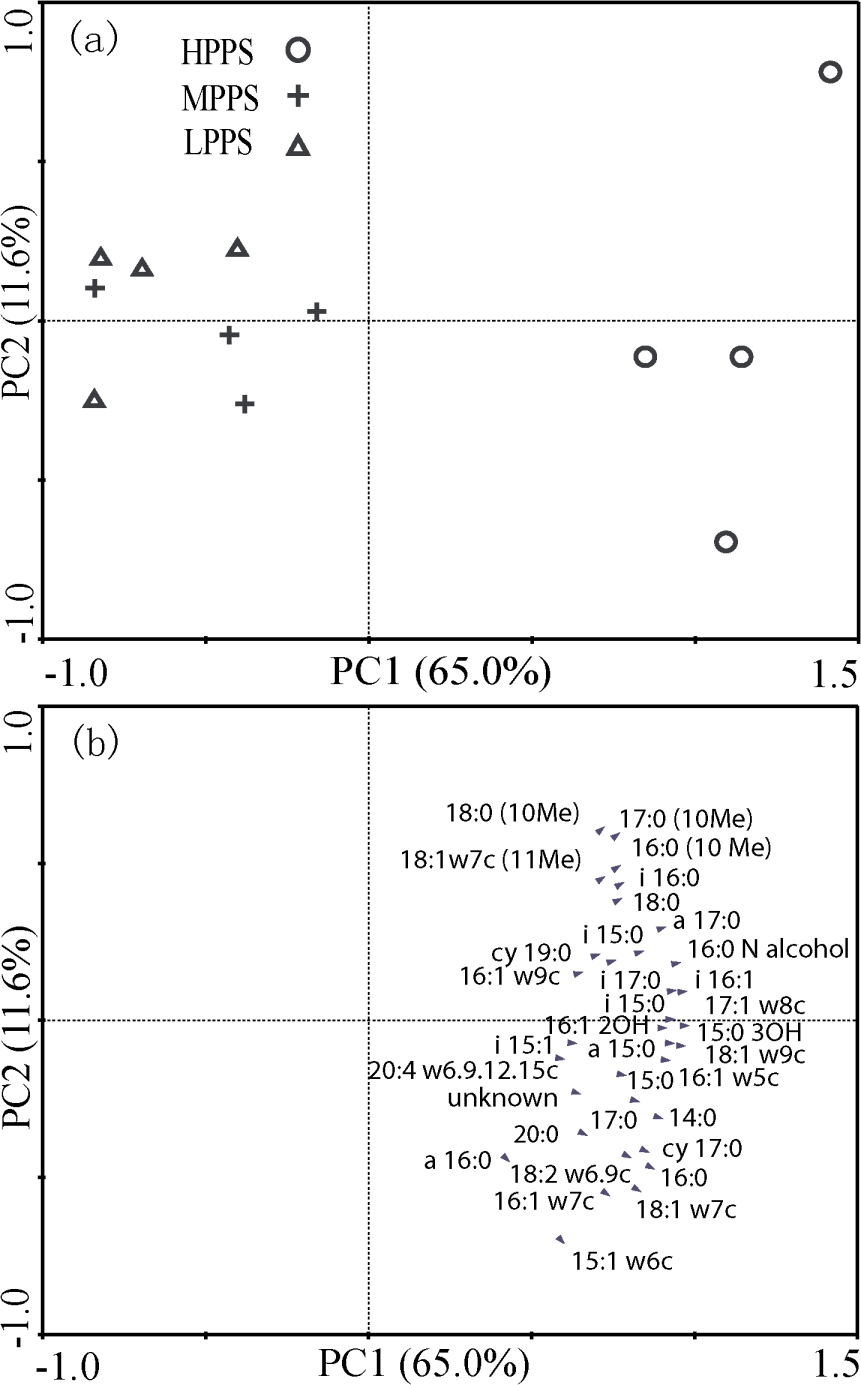
Plot of the first two principal components (PC1 and PC2) grouped in HPPS, MPPS and LPPS (a) and plot of the PC1 and PC2 for 35 PLFAs (b).

Canonical analysis of the PLFA data indicated that the microbial community structures of each productivity class of waterlogged purple paddy soil showed only small differences among the four typical locations, and HPPS were well separated from MPPS and LPPS (Fig 2A). The PC1 and PC2 accounted for 65.0% and 11.6% of the total variation, respectively, whereas the PC loadings of each PLFA showed that almost all PLFA bioindicators were characterized by high concentrations in HPPS (Fig 2B).

Microbial indicators can serve as early and sensitive markers of soil degradation or improvement [55], and also can indicate the degree of soil fertility and the quality of agricultural management [56]. MPPS and LPPS characterized by relatively low PLFA concentrations were associated with poor soil fertility and quality. In this way, manure amendment could be carried out because of its beneficial to improve soil biofertility and quality in purple soil [57].

### Correlation analysis

Correlation analysis was conducted with 24 parameters including soil properties and rice yield (Table 4). Rice yield was significantly correlated with all measured soil parameters except for AN, AK and dehydrogenase. The high levels of AN and its non-significant correlation with rice yield suggested that AN is not the factor limiting crop production. The significant correlations between selected soil microbial communities and rice yield as well as their relatively high correlation coefficients support the conclusion established by Lima et al. (2013) [12], who reported that biological indicators are the most sensitive in indicating differences in soil quality under rice production systems. SOC and TN were considered as the two most important soil chemical parameters, because they markedly affected soil enzymes (urease, dehydrogenase, β-glucosidase, arylsulphatase and peroxidase), biological properties (total PLFA, gram-positive, gram-negative, fungi and AMF) and several other chemical properties (CEC, AZn). The results followed the observations of Bonanomi et al. (2014) [54], who found that high enzyme activities were mainly attributable to the relatively higher SOC and next to energy sources and nutrients that sustain growth and activity of soil microbes.

**Table 4:** Correlation coefficients among rice yield and soil physico-chemical and biological properties.

	Yield	BD	pH	SOC	TN	AN	AP	AK	CEC	ASi	AZn	Urease	DH	BG	ARS	ACP	POD	PLFAs	G+	G-	TB	ACT	Fungi	AMF
Yield	1.00																							
BD	-0.83	1.00																						
pH	-0.60	0.53	1.00																					
SOC	0.47	-	-	1.00																				
TN	0.63	-0.55	-0.47	0.75	1.00																			
AN	-	-	-	-	-	1.00																		
AP	0.56	-0.39	-	0.34	0.36	-	1.00																	
AK	-	-	-	-	-	-	-	1.00																
CEC	-0.65	-	0.59	-0.74	-0.59	-	-0.50	-	1.00															
ASi	0.49	-0.38	-	-	-	-	0.45	-	-	1.00														
AZn	0.59	-0.59	-	0.62	0.72	-	0.61	-	-0.36	-	1.00													
Urease	0.69	-0.67	-0.48	0.39	0.48	-	0.63	-	-0.48	0.48	0.52	1.00												
DH	-	-	-	0.53	0.34	-	0.49	-	-0.64	0.42	-	-	1.00											
BG	0.65	-0.49	-0.69	0.64	0.61	-	-	-0.35	-0.72	0.37	0.39	0.42	0.44	1.00										
ARS	0.46	-	-0.48	0.75	0.53	-	0.52	-	-0.85	0.34	0.46	0.40	0.82	0.64	1.00									
ACP	0.46	-0.48	-0.34	-	-	-	-	0.44	-	0.35	-	-	-0.35	-	-	1.00								
POD	0.39	-	-	0.64	0.49	-	-	-	-0.56	-	-	0.33	0.55	0.55	0.61	-	1.00							
PLFAs	0.69	-0.68	-0.51	-	0.52	-	-	-	-0.37	0.39	0.35	0.49	-	0.58	0.34	0.43	0.47	1.00						
G+	0.65	-0.66	-0.46	-	0.43	-	-	-	-	0.47	-	0.39	-	0.62		0.47	0.45	0.95	1.00					
G-	0.65	-0.46	-0.48	0.82	0.77	-	0.49	-	-0.75	-	0.60	0.47	0.43	0.58	0.68	-	0.54	0.55	0.43	1.00				
TB	0.77	-0.63	-0.55	0.68	0.72	-	0.38	-	-0.67	0.41	0.52	0.49	0.39	0.73	0.60	-	0.59	0.86	0.81	0.87	1.00			
ACT	0.51	-0.59	-0.49	-	-	-	-	-	-	0.33	-	0.42	-	0.47	-	0.37	-	0.93	0.92	-	0.68	1.00		
Fungi	0.71	-0.51	-0.37	0.73	0.77	-	0.46	-	-0.69	-	0.63	0.35	0.42	0.69	0.58	-	0.59	0.56	0.57	0.76	0.82	0.33	1.00	
AMF	0.61	-0.41	-0.49	0.40	-	-	0.63	-	-0.65	0.72	-	0.58	0.67	0.55	0.77	-	0.47	0.66	0.62	0.58	0.69	0.58	0.43	1.00

Only the significant correlations are shown.

Abbreviations: BD, bulk density; SOC, soil organic carbon; TN, total N; AN, available N; AP, available P; AK, available K; CEC, cation exchange capacity; ASi, available Si; AZn, available Zn; DH, dehydrogenase; BG, β-glucosidase; ARS, arylsulfatase; ACP, acid phosphatase; POD, peroxidase; G+, gram-positive; G-, gram-negative; TB, total bacteria; ACT, actinomycetes; AMF, arbuscular mycorrhizal fungi.

As presented in Table 4, total PLFAs was significantly correlated with most soil enzymes, and similar findings were also reported by Giacometti et al (2013) [9]. In addition, soil pH strongly affects soil enzyme activities (i.e. urease, β-glucosidase, arylsulphatase, acid phosphatase), and negative correlations were observed between pH and PLFA bioindicators. This is consistent with the findings of Zhou et al. (2014) [58], who concluded that soil pH might have an essential role in controlling soil microbial structure in purple soil. In addition, soil CEC was signficantly and positively correlated with pH, and the low CEC in HPPS may attribute to its soil acidification [34]. The negative correlations between rice yield and pH/CEC suggested that low levels of pH and CEC may be potential constrants limiting the productivity in HPPS.

### Soil quality assessment

Based on correlation analysis results (Table 4), variables of AN, AK and dehydrogenase were not well correlated with rice yield, suggesting that those soil properties were not limiting or enhancing the rice growth. Therefore, AN, AK and dehydrogenase were excluded from soil quality evaluation in this study. Additionally, ASi and AZn were also omitted because of their concentrations typically higher than the critical values, although they presented significant correlations with rice yield. Ultimately, BD, pH, SOC, TN, AP, CEC, Urease, β-glucosidase, arylsulphatase, acid phosphatase, peroxidase, total PLFAs, G+, G-, TB, actinomycete, fungi and AMF were selected as optimal soil quality indicators for PCA.

The PCA showed that more than 82% of the variance in the data was explained by the first four PCs with Eigenvalues ≧ 1 (Table 5). The highly weighted variables under PC4, defined as those within 10% of the highest weight of the factor loading were total N and AMF. Both total N and AMF were selected for the MDS because they were not highly correlated (r = 0.31). In PC1, PC2 and PC3, only one highly weighted variable was observed. Therefore, total bacteria, acid phosphatase and AP were all retained in the MDS and the refined MDS was established including TN, AP, acid phosphatase, total bacteria and AMF.

**Table 5:** Results of principal components analysis (PCA) of statistically significant soil quality indicators.

Soil quality attribute	PC1	PC2	PC3	PC4
Eigenvalues	9.498	2.827	1.407	1.043
% of variance	52.77	15.71	7.81	5.79
Cumulative percent	52.77	68.47	76.29	82.08
Factor loading				
Bulk density	-0.682	-0.438	0.181	0.402
pH	-0.661	-0.202	0.219	0.026
Soil organic C	0.730	-0.562	0.175	-0.159
Total N	0.769	-0.215	0.131	**-0.458**
Available P	0.526	-0.211	**-0.696**	0.006
CEC	-0.792	0.414	0.127	-0.125
Urease	0.658	0.095	-0.514	-0.106
Glucosidase	0.815	-0.059	0.207	0.051
Phosphatase	0.186	**0.736**	-0.225	-0.173
Arylsulfatase	0.733	-0.502	-0.097	0.339
Peroxidase	0.652	-0.289	0.312	0.207
PLFAs	0.801	0.514	0.185	0.099
Gram-positive	0.750	0.546	0.284	0.143
Gram-negative	0.846	-0.261	-0.001	-0.184
Total bacteria	**0.948**	0.103	0.173	-0.033
Actinomycetes	0.628	0.655	0.184	0.229
Fungi	0.818	-0.223	0.214	-0.239
AMF	0.769	0.08	-0.299	**0.504**

Boldface numbers are heavily weighted factors under each principal component (PC).

**Fig 3:**
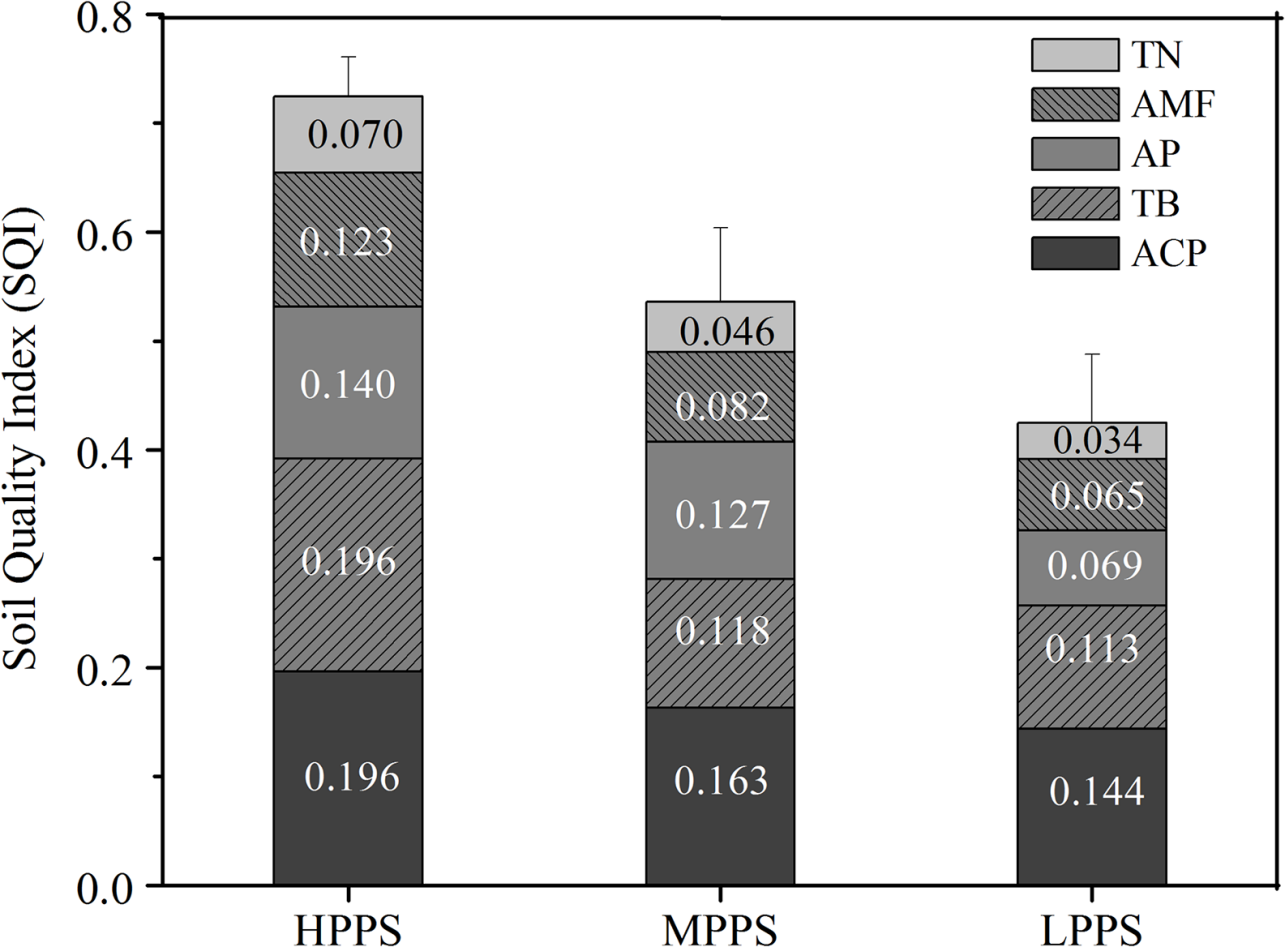
Soil quality indices (SQIs) of HPPS, MPPS and LPPS. Stacked bars represent the index values for weighted MDS variable scores. Error bars denote standard deviation of overall index values.

After the MDS selection process, an additional PCA was conducted with MDS indicators. The weighting factors for TN, AP, acid phosphatase, total bacteria and AMF were 0.186, 0.142, 0.253, 0.222 and 0.197, respectively. Each MDS variable was transformed to a value between 0 and 1 using their individual scoring functions. A standard scoring function was used to normalize TN and AP as described in Qi et al. (2009) [14]. The remaining MDS variables, phosphatase, total bacteria and AMF, were considered as "higher is better" indicators and were transformed using a linear scoring function [30]. Finally, SQI was calculated using the integrated quality index equation (Eq (1)). The soil quality indices of the selected waterlogged purple paddy fields varied from 0.295 to 0.794. The mean SQI was highest for HPPS (0.725±0.046) followed by MPPS (0.536±0.068) and LPPS (0.425±0.063) (Fig 3). While the HPPS represented the highest soil quality, the LPPS represented the poorest soil quality status, and low levels of TN and AP were found to be its major constraints limiting rice productivity. High soil quality can maintain high productivity without significant soil or environment degradation [59]. Mangers in our study area should pay more attention to the LPPS and particularly to any special limiting factor.

**Fig 4:**
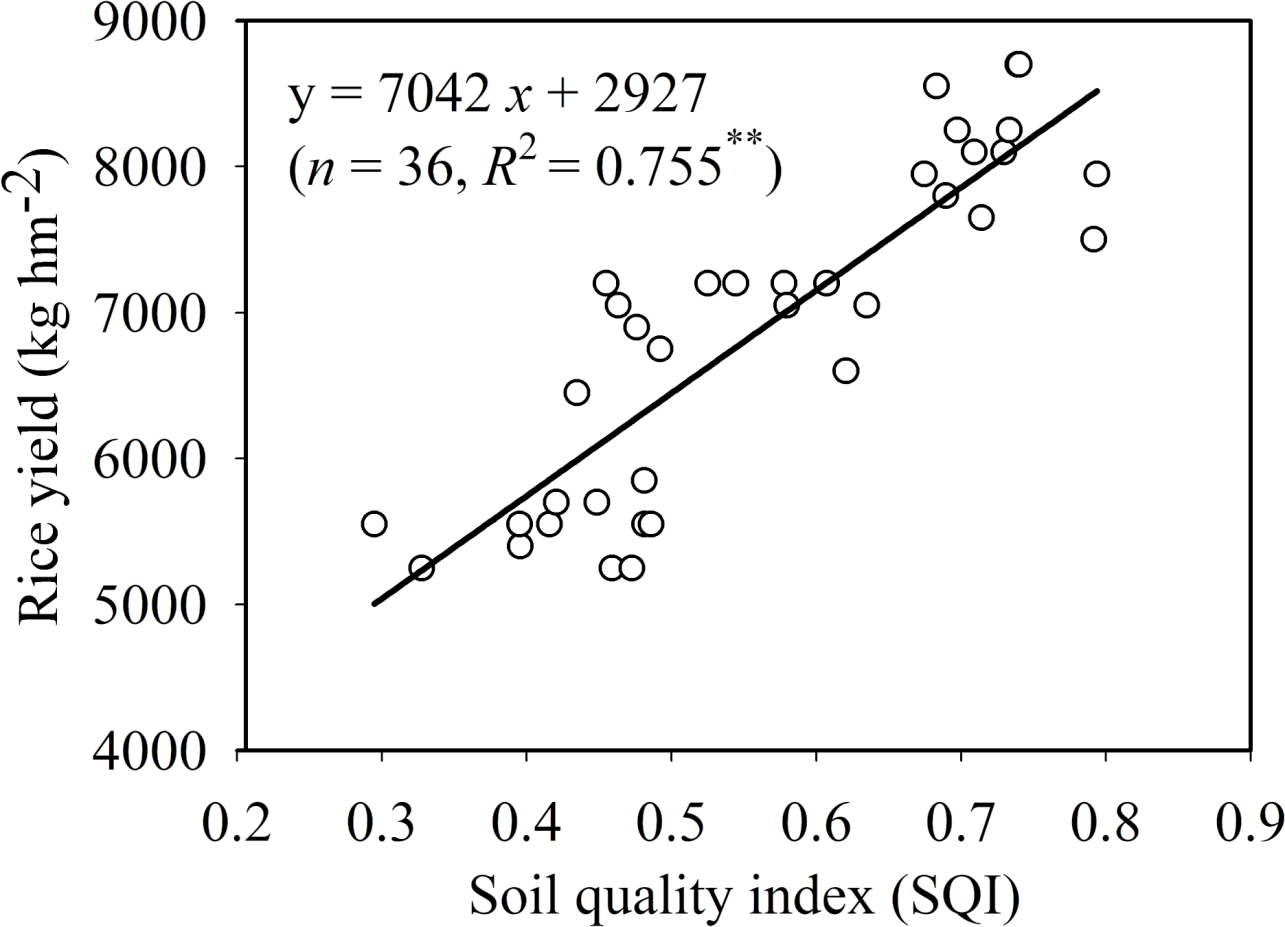
Correlation between soil quality index and rice yield of 36 paddy fields. ^**^ P < 0.01.

Correlation analysis indicated that SQI and rice yield were linearly related (Fig 4). The results followed the observations of Li et al. (2013) [17], who also reported significant positive correlation between SQI and ric yield. Our results also support previous conclusions that the selected MDS indicators could effectively evaluate soil quality status as a rice-production medium and the SQI approach is an appropriate way to develop a quantitative procedure to evaluate different land management practices [11,12].

## Conclusion

An MDS including TN, AP, acid phosphatase, total bacteria and AMF was established for evaluating soil quality of waterlogged paddy soil in acidic purple soil region. SQI was also created based on the MDS method, and HPPS, MPPS and LPPS received mean SQI scores of 0.725, 0.536 and 0.425, respectively. LPPS presented a worst soil quality status, and low levels of TN, AP and soil microbial activities were considered to be the primary constraints limiting its productivity compared to HPPS. All soil samples collected were rich in available N, K, Si and Zn, but deficient in available P, which may be the major constraint for the studied regions. Based on our results, a higher dose of phosphorus fertilizer should be considered to prevent severe deficiency of AP while the rate of basic N fertilizer should be decreased, with the saved N fertilizer to be applied as additional topdressing fertilizer. Other effective measures (i.e. soil-biochar additions, manure amendment) could be conducted because of their beneficial to counteract soil degradation [60] and improve soil biological quality [61], and future researches are resquired to confirm those hypothesises.

## Supporting Information

S1 Data The relevant data used for all Figures. (XLSX)
